# A new member of troglobitic Carychiidae, *Koreozospeum nodongense* gen. et sp. n. (Gastropoda, Eupulmonata, Ellobioidea) is described from Korea

**DOI:** 10.3897/zookeys.517.10154

**Published:** 2015-08-12

**Authors:** Adrienne Jochum, Larisa Prozorova, Mariana Sharyi-ool, Barna Páll-Gergely

**Affiliations:** 1 Naturhistorisches Museum der Burgergemeinde Bern, CH-3005 Bern, Switzerland, Institute of Ecology and Evolution, University of Bern, 3012 Bern, Switzerland; 2 Institute of Biology and Soil Science, Far Eastern Branch of Russian Academy of Sciences, Vladivostok, 690022, Russia; 3 Department of Biology, Shinshu University, Matsumoto 390-8621, Japan

**Keywords:** Cave-dwelling species, subterranean snail, energy-dispersive X-ray spectrometry, microgastropoda, ecology, conservation

## Abstract

A new genus of troglobitic Carychiidae Jeffreys, 1830 is designated from Nodong Cave, North Chungcheong Province, Danyang, South Korea. This remarkable find represents a great range extension and thus, a highly distant distribution of troglobitic Carychiidae in Asia. The *Zospeum*-like, carychiid snails were recently included, without a formal description, in records documenting Korean malacofauna. The present paper describes *Koreozospeum* Jochum & Prozorova, **gen. n.** and illustrates the type species, *Koreozospeum nodongense* Lee, Prozorova & Jochum, **sp. n.** using novel Nano-CT images, including a video, internal shell morphology, SEM and SEM-EDX elemental compositional analysis of the shell.

## Abstract (kr)

Carychiidae Jeffreys, 1830 (양귀비고둥/동굴거주생명체, 제프리 1830) 의 새로운 속은 한국 충청북도 단양의 노동동굴에서 지정되었다. 이 놀라운 발견은 이 동굴거주생물의 아시아지역까지의 광대한 확장과 상당히 분리된 분포를 나타낸다. “*Zospeum*-like” (유럽에서 발견된 달팽이종), carychiid snails 은 최근에 공식적인 명칭없이 한국의 연체동물문서목록에 포함되었다. 본 논문은*Koreozospeum* Jochum & Prozorova, **gen. n.** 을 설명하고 껍질내부 X -ray촬영, SEM과 SEM-EDX 껍질 기본 구성분석등을 나타내는 영상을 포함, 첨단 나노-CT 이미지를 이용하며 신종 *Koreozospeum nodongense* Lee, Prozorova & Jochum, **sp. n.** 을 묘사한다.

## Introduction

**Figure 1.**
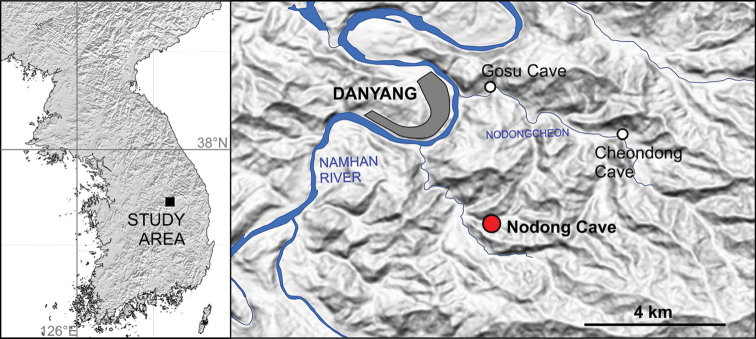
Map indicating location of Nodong cave (locus typicus), Danyang, North Chungcheong Province, South Korea. Red dot, *Koreozospeum nodongense* sp. n.; White dots indicate potential *Koreozospeum nodongense* sp. n. habitats in Gosu and Cheondong caves in the vicinity of Nodong cave.

**Figure 2.**
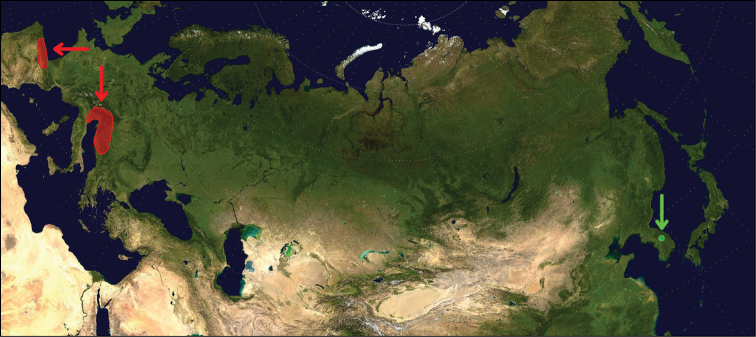
Map indicating the extreme distant distribution of subterranean Carychiidae represented by *Koreozospeum nodongense* sp. n. (Nodong cave, South Korea, locus typicus) in conjunction with the known subterranean distribution of European *Zospeum* Bourguignat, 1856 in Northern Spain, the Southern Alps and the Dinaric Alps. Red colour indicates *Zospeum*, and green indicates *Koreozospeum*.

It is estimated that the Korean peninsula harbors more than 1,000 caves within its Cambro-Ordovician limestone geology (Kashima et al. 1978, Woo et al. 2001). Of these caves, only one, Nodong-donggul (Nodong cave, 36°57.186'N, 128°22.938'E) in North Chungcheong Province, South Korea (Fig. 1) is so far known to contain finds of “*Zospeum*-like” carychiid microgastropods (Kwon et al. 2001, Lee and Min 2002, Min et al. 2004). The shell shape and microsculpture of these tiny snails most closely resemble the troglobitic genus *Zospeum* Bourguignat, 1856 (Ellobioidea, Carychiidae) rather than epigeal *Carychium* O. F. Müller, 1774 (Prozorova et al. 2010, 2011). Cave-dwelling species are not known from nearby Japan, which was recently found to contain the highest lineage diversity for Carychiidae Jeffreys, 1830 (Weigand et al. 2013a). The present material comprises the first account of troglobitic Carychiidae in Asia. Up to now, subterranean taxa included only members of the genus *Zospeum*, exclusively known to inhabit karst caves of southern Alpine Europe (Jochum et al. 2015). (The North American species, *Carychium stygium* Call, 1897 is no longer considered an exclusively troglobitic species (Weigand et al. 2011, 2013b)). The taxon described here represents an extreme range extension to Asia for subterranean ellobioid snails (Fig. 2).

Open to the public as a tourist attraction, Nodong cave extends approximately 800 m in length and drops 300 m in vertical depth. Geographically, it is located near the Nodongcheon, a branch of the Namhan River (Lee 2012) and near the city of Danyang, a resort town at the base of the extensive Sobaeksan National Park. Other known caves and potential habitats for troglobitic carychiid snails in the immediate vicinity include the public caves, Gosu and Cheondong.

When material, such as the shells of troglobitic carychiids, is particularly limited and rare, contemporary non-destructive techniques for taxonomic assessment are essential. Applied in taxonomy, contemporary methods used primarily in medicine and industry can provide new opportunities for understanding global and local biodiversity. They can also act as catalysts for igniting dire conservation strategies regarding rare populations and for extracting valuable information sequestered in their organic forms. In this work, one of six known Korean carychiid shells has been examined using Nano-CT imaging to assess and compare the internal shell morphology of *Koreozospeum nodongense* sp. n. with its supposed closest relative, the European genus *Zospeum*. In addition, available shell fragments of *Koreozospeum nodongense* sp. n. material were examined via scanning electron microscopy (SEM) coupled with energy-dispersive X-ray spectrometry (EDX) to investigate the internal morphology of the shell and to determine the elemental composition of the shell matrix. In addition, and as a secondary consideration, limited information is available regarding the specific geology and ecology of Nodong cave and adjacent, potentially contiguous caves (i.e. Gosu cave and Cheongong cave) of North Chungcheong Province. SEM-EDX elemental compositional analysis opens windows for inference about the subterranean ecology of *Koreozospeum nodongense* sp. n. and likely the ecology of adjacent caves for future investigation.

In this work, a new subterranean taxon is we described from Korea based on characters significantly differentiating from European *Zospeum* morphotypes. SEM and Nano-CT images of the intact shell of the new species and SEM-EDX graphic images of the elemental composition of selected sections of shell fragments are presented.

## Material and methods

Similar to conditions known for *Zospeum* (see Jochum et al. 2015), carychiid snails were collected live on muddy walls in January 2000 by J.-S. Lee in the dark zone of Nodong cave (Prozorova et al. 2010, 2011).

One shell (Holotype NMBE 534197/1) available for examination outside of Vladivostok and Korea (99 lost, see below) and six paratypes located in Vladivostok were measured according to Jochum et al. (2015, fig. 1). The number of whorls was counted according to the method described in Kerney and Cameron (1979). For the species description, shell measurements are expressed as: shell height (SH); shell width (SW); height of the last whorl (HLWH); peristome height (PH); peristome diameter (PD); spire Angle (SA); number of whorls (W); widest diameter (WD) (distance from top to bottom). Spire angle (SA) is given in degrees. Other measurements are in mm. Measurements of the holotype (NMBE 534197/1) were taken from images obtained using a Leica DFC420 digital camera attached to a Leica M165c stereo microscope, supported by Leica LAS V4.4 software. Measurements of the paratypes (ZIN RAS 1) were taken using the LOMO MBS-10 stereo microscope (Lytkarino, Ru.). Qualitative aspects of shell morphology including peristome shape; whorl profile (whorl convexity); protoconch and teleoconch sculpture; description of the lamella on the parieto-columellar region of the aperture; configuration of the columellar lamella and the independent configuration of the columella are documented.

Since the individuals reported by Prozorova et al. (2011), which were housed in the Min Molluscan Research Institute in Seoul, South Korea have become regretfully lost to science, as much information as possible was extracted from the holotype (NMBE 534197/1), one paratype (IBSS FEB RAS 7787) and some fragments (paratype NMBE 534361/2) using Nano-CT imaging (whole shell), SEM and SEM-EDX energy-dispersive X-ray spectrometry (fragments). No individuals were preserved in ethanol, precluding molecular analyses and anatomical examination.

### Image acquisition

SEM: *Koreozospeum nodongense* sp. n. (IBSS FEB RAS 7787 paratype) (now damaged) was coated with carbon and imaged (Prozorova et al. 2011) at the Centers of Collective Use in IBSS and the Institute of Marine Biology FEB RAS using the Zeiss EVO −40 scanning electron microscope (Jena, Germany) implementing the Variable Pressure (VP) mode.

SEM-EDX: Morphological (SEM) and elemental composition (EDX) of *Koreozospeum nodongense* sp. n. paratype (NMBE 534361/2) fragments were assessed using the FEI-Aspex Explorer scanning electron microscope system (Hillsboro, OR, USA), implementing a BE detector for image generation. Non-coated shell material was placed on a cellulose membrane and mounted on a computer-controlled stage for scanning. Elemental composition was detected (i.e. each element shows a multiple-peak pattern in the spectrum) by using an emission current of 29 mA, an electron beam acceleration voltage of 20 kV under sample pressure of 0.15 Torr and a working distance of 22.9 mm at RJL Micro & Analytic GmbH, Karlsdorf-Neuthard, Germany. In our analyses, some peaks overlap, whereby the elemental letters also overlap. Peak height represents the intensity of the element and this is proportional to the mass percentage present in the assessed shell region.

Micro-CT: *Koreozospeum nodongense* sp. n. (NMBE 534197/1) was imaged using a nano-computed tomography system (Nano-CT), manufactured and developed by Bruker-Micro-CT/SkyScan (SkyScan 1172, Kontich, Belgium). The video of *Koreozospeum nodongense* sp. n. was created using a SkyScan 1172 scanner at RJL Micro & Analytic GmbH, Karlsdorf-Neuthard, Germany. The scanner is equipped with a sealed micro focus X-ray source and a 11 Mpx CCD detector. The specimen was scanned with 4 µm voxel size in rotation steps of 0.6° at 59 kV tube voltage and 167 µA tube current. Reconstruction with cross sectional images was performed using a modified Feldkamp cone-beam reconstruction algorithm. Image resolution of the cross sectional images was 4 µm isotropic voxel side length with a grey scale resolution of 8 bit. The animated video was generated using a direct volume rendering method implemented in the software CTvox.

Digital images: *Koreozospeum nodongense* sp. n. (holotype NMBE 534197/1) and fragments of the ultimate whorl (paratype NMBE 534361/2) were photographed using a Leica DFC 425 multilayered photography system. All measurements are in mm.

### Abbreviations

ANSP Academy of Natural Sciences, Philadelphia, Pa., USA

IBSS FEB RAS Institute of Biology and Soil Science, Far Eastern Branch of Russian Academy of Sciences, Vladivostok, Russia

MHNG Museum d’Histoire Naturelle de Genève, Geneva, Switzerland

MMRI Min Molluscan Research Institute, Seoul, South Korea

MNCN Museo Nacional de Ciencias Naturales, Madrid, Spain

NHMUK Natural History Museum, London, UK

NHMW Naturhistorisches Museum, Wien, Austria

NMBE Naturhistorisches Museum der Burgergemeinde Bern, Switzerland

ZIN RAS Zoological Institute of the Russian Academy of Sciences, St. Petersburg, Russia

ZUPV/EHU Colección de Fauna Cavernícola (Departamento de Zoología) de la Universidad del País Vasco-Euskal Herriko Unibertsitatea, Bilbao, Spain

## Taxonomy

### Family Carychiidae Jeffreys, 1830

#### Genus Koreozospeum

Taxon classificationAnimaliaPulmonataCarychiidae

Jochum & Prozorova gen. n.

http://zoobank.org/FDA3DA2E-7FEE-4C65-ACFF-D8C953E1A2CA

Figures 3 , 4 , 5 , 6 , 7 , 8 , 9 , 10 , 11

##### Type species.

*Koreozospeum nodongense* sp. n.

##### Diagnosis.

Shell thin, ovate-conic, fine spiral rows of interconnected pits constant throughout teleoconch, peristome oblique auriform, conspicuous plicate lip (side view).

##### Differential diagnosis.

Differs from *Carychium* by its squat ovate-conic form, absence of major apertural dentition and its singularly troglobitic ecology; from *Zospeum* by the oblong, slightly detached, oblique, auriform peristome, shallow suture, minimally convex whorls, interrupted low lamella on roof of interior penultimate whorl forming annular lamella, and the conspicuously pleated lip folded back onto the body whorl and not rolled into the body whorl as in *Zospeum*.

##### Derivatio nominis.

The name derives from Korea, the land of the type locality and the similarity to European *Zospeum*.

##### Distribution.

Only known from Nodong cave.

#### Koreozospeum nodongense

Taxon classificationAnimaliaPulmonataCarychiidae

Lee, Prozorova & Jochum sp. n.

http://zoobank.org/F740D7E3-6C8D-4A0E-AD62-0FBDBE6A042D

Figures 3 , 4 , 5 , 6 , 7 , 8 , 9 , 10 , 11

“Carychium” sp. Min, Lee, Koh and Je, Mollusks in Korea. Min Molluscan Research Institute, Seoul, Korea. 566 pp., 342−343, fig. 1080. 2004
“Zospeum” sp. Prozorova, Lee and Zasypkina, Korean Journal of Soil Zoology 15(1−2): 1−4, figs 1–3. 2011

##### Material.

*Type material*. Holotype (NMBE 534197/1): South Korea, North Chungcheong Province, Danyang County, Nodong cave, 36°57.186'N, 128°22.938'E, alt. ca. 271 m, moist muddy walls in cave, 13.01.2000, leg. Jun-Sang Lee.

Paratypes: *locus typicus*: 3 fragments (NMBE 534361/2), data as the holotype; 1 broken shell (IBSS FEB RAS 7787), ibid.; 5 shells, 1 broken (ZIN RAS 1), ibid.

##### Diagnosis.

Shell small, thin, ovate-conic, smooth, fine spiral rows of interconnected pits constant throughout teleoconch, plicate apertural lip may or my not be present (side profile).

##### Description.

**Figure 3.**
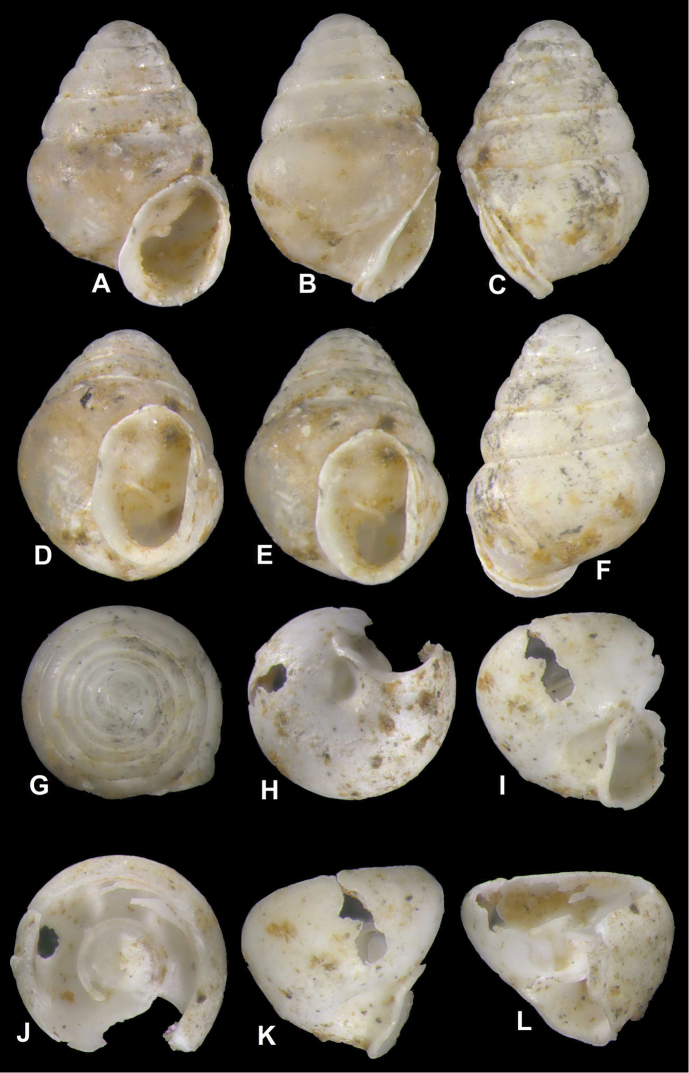
*Koreozospeum nodongense* sp. n., **A–G** different views of holotype (NMBE 534197/1) **H–L** different views of body whorl fragment of paratype (NMBE 534361/2) **H** umbilical notch **I, K** side view of umbilical region **J** areal view of columella surrounded by the single, low annular lamella **L** side view of lamella and orientation to the columella.

**Figure 4.**
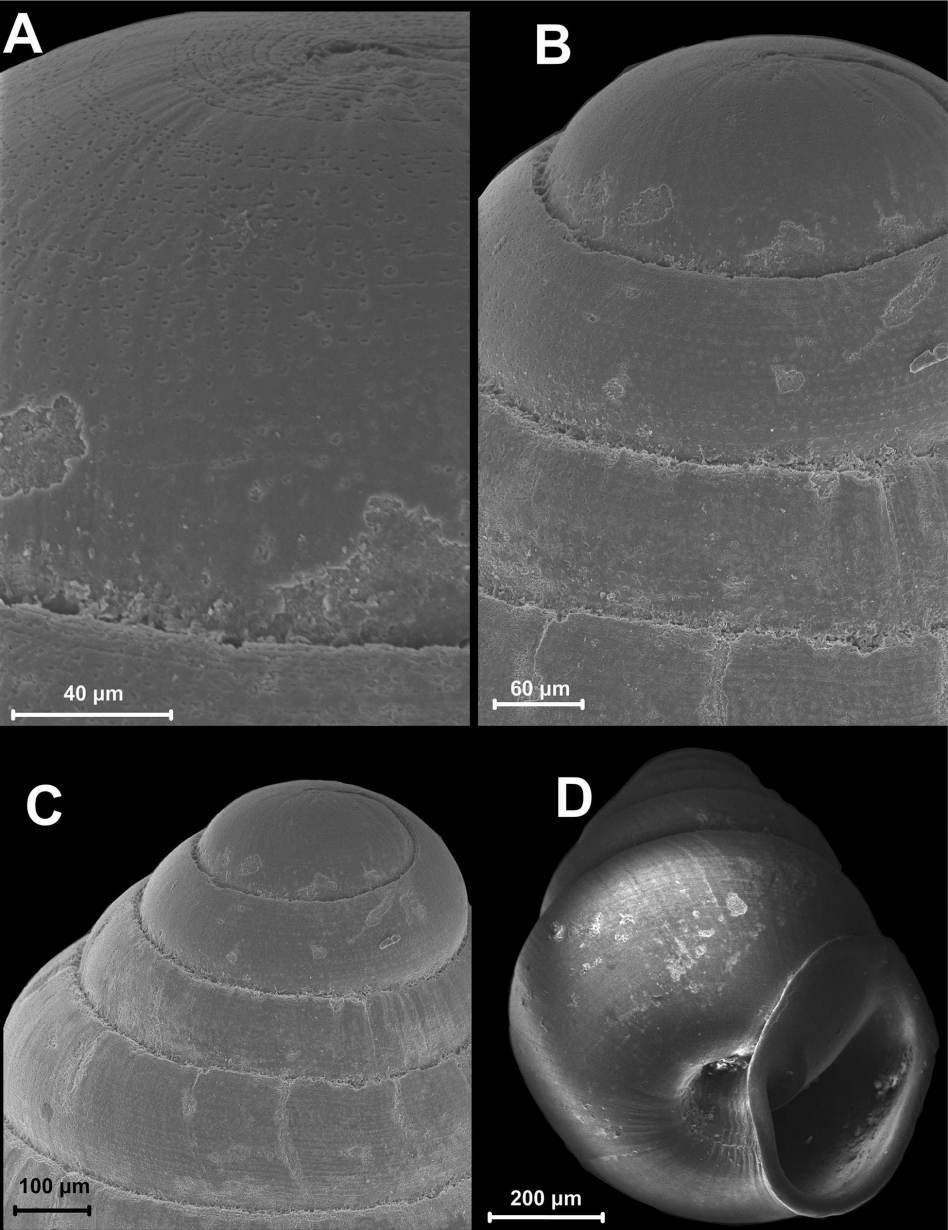
SEM of paratype (IBSS FEB RAS 7787). **A** Protoconch and **B–C** apical whorls showing pitted pattern of microstructure and shallow suture **D** umbilical notch showing wrinkles behind the peristome. Scale bar increments µm.

**Figure 5.**
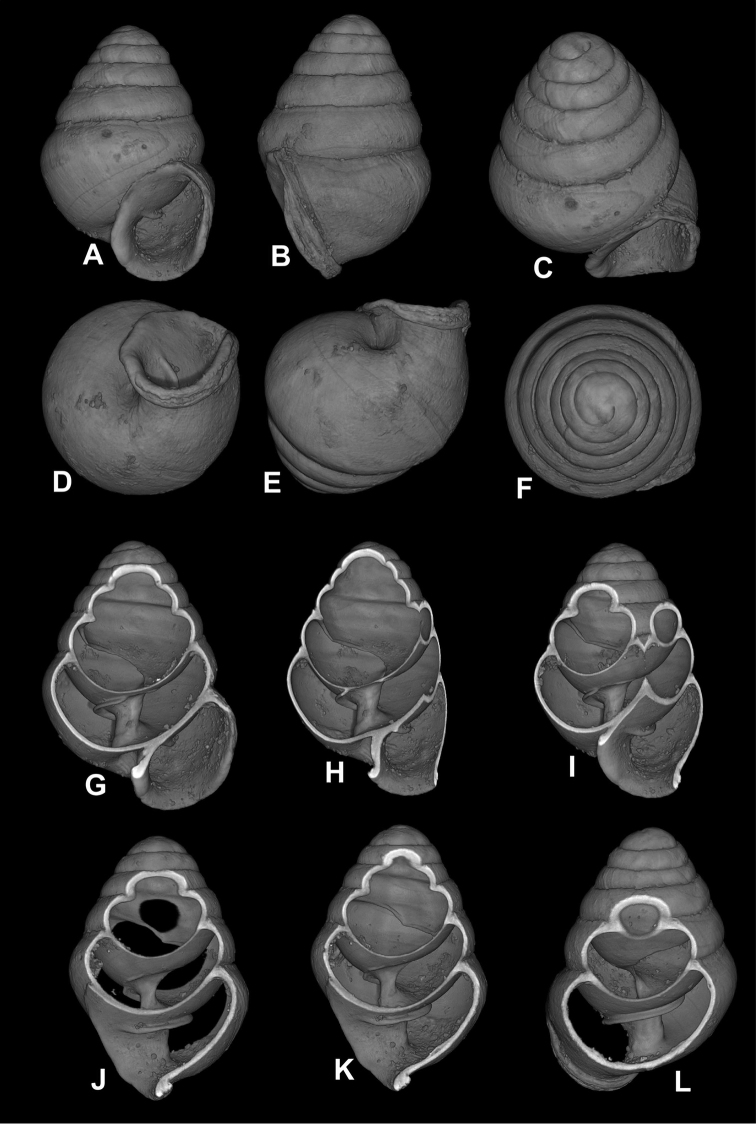
Nano-CT images of *Koreozospeum nodongense* sp. n., **A–F** different views of holotype (NMBE 534197/1) **G–L** cross sections showing shell interior **G–H** clavate columella **I–L** annular lamella.

*Koreozospeum nodongense* sp. n. is characterized by a very small, alabastrine, ovate conical shell with 5 regular, moderately increasing whorls. The penultimate whorl is slightly angularly shouldered at the uppermost extension of the peristome in left and right profile positions (Fig. 3B–C). Peristome oblong, auriform, oblique to shell axis, partially adnate to ultimate whorl, otherwise slightly detached (Fig. 3K), more or less thickened (Fig. 3A, D–E); the lip is folded back onto the body whorl and thickly plicate 3/4 of the lip side-view height (Figs 3C, 4B, E); deep umbilical notch (Figs 3 H–I) with wrinkles projecting into notch behind peristome region (Fig. 4D); robust columellar lamella running into the shell interior (Figs 3A, D–E, 5I–K). The protoconch is obtuse and shows a pattern of spiral interconnected pits (Fig. 4); the teleoconch bears tightly spaced irregular spiral striae of densely interconnected pits (Figs 4, 9) and shows a marbled surface pattern of faint, horizontally-elongated chevrons intercalating with each successive whorl (Fig. 5 A, C). Suture irregular and shallow, bordered by white marginal zone at each increasing abapical whorl (Figs 3C, F, 4). Interior perspectives show a parietal structure consisting of a partially discontinuous lamellar ridge on the roof of the penultimate whorl (Fig. 4I–K), which then develops into the uniformly shaped annular lamella running directly under the penultimate whorl into the aperture. The columella is moderately slender, clavate (Fig. 5G–H) with a single, annular lamella (Fig. 5G–L).

##### Measurements

(in mm). Holotype (NMBE 534197/1) (Figs 3A–G, 7): H = 1.72; SW = 1.19; HLWH = 1.13; PH = .78; PD = .69; SA = 68.6; W = 5.65; WD = .81. See also Table 1.

**Figure 6.**
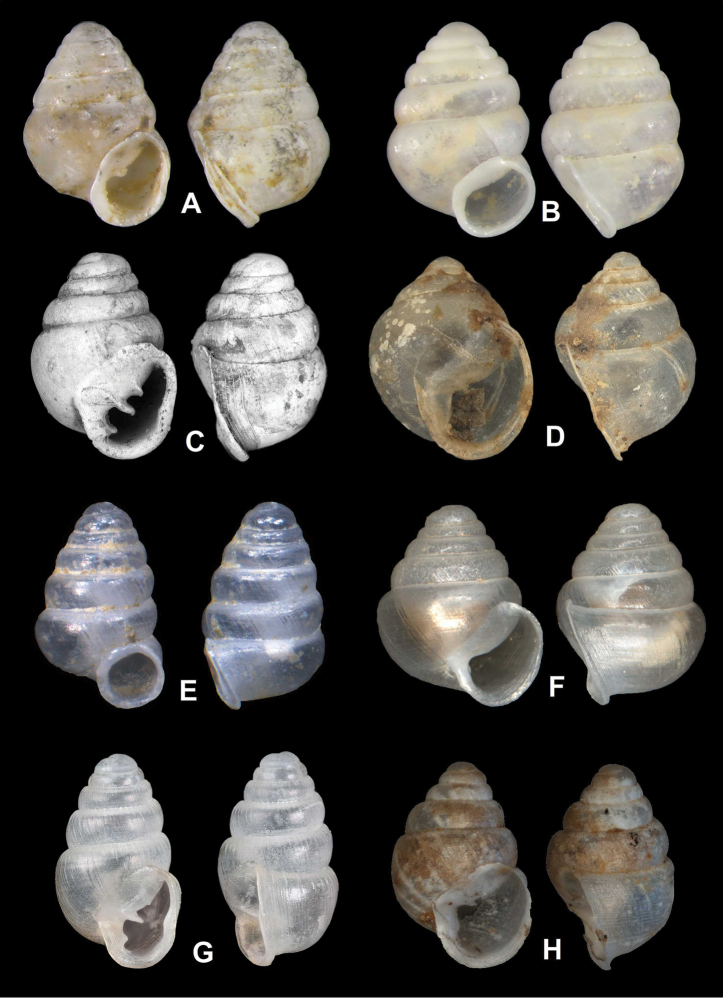
Comparative images of European *Zospeum* Bourguignat, 1856. Museum type material showing ventral and side views. **A** *Koreozospeum nodongense* sp. n. Holotype (NMBE 534197/1) **B** *Zospeum bellesi* E. Gittenberger, 1973 (Syntype ZUPV/EHU 188) **C** *Zospeum lautum* (Frauenfeld, 1854) (Holotype ANSP 22529); **D** *Zospeum obesum* (Frauenfeld, 1854) (Syntype MHNG 7904) **E** *Zospeum vasconicum* Prieto, De Winter, Weigand, Gómez & Jochum, 2015 (Holotype MNCN15.05/60147H) **F** *Zospeum zaldivarae* Prieto, De Winter, Weigand, Gómez and Jochum, 2015 (Holotype MNCN15.05/60148H); **G** *Zospeum spelaeum schmidtii* (Frauenfeld, 1854) (Syntype NHMUK 1991027) **H** *Zospeum exiguum* Kuščer, 1932 (Holotype NHMW 32008).

**Figure 7.**
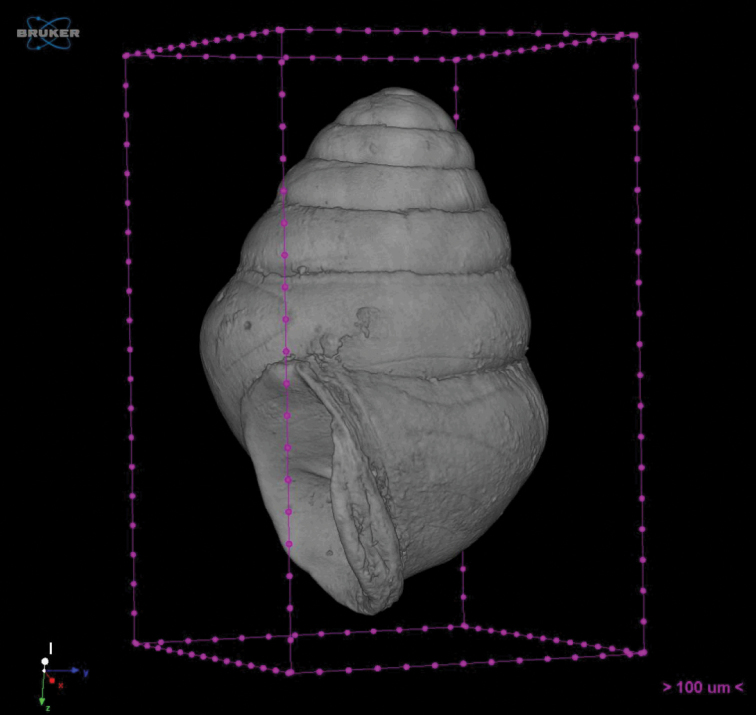
Nano-CT film of *Koreozospeum nodongense* sp. n. holotype (NMBE 534197/1) YouTube link: https://youtu.be/SU020_GmLaA.

**Table 1.** Measurements of six paratypes (ZIN RAS 1) and holotype (NMBE 534197/1) of *Koreozospeum nodongense* sp. n., including shell condition (SC) and apertural lip configuration (ALC) in side view. Measurements in mm. Spire angle (SA) in degrees.

	Holotype NMBE 534197/1	P-type 1 ZIN RAS 1	P-type 3 ZIN RAS 1	P-type 4 ZIN RAS 1	P-type 5 ZIN RAS 1	P-type 6 ZIN RAS 1	P-type 2 IBSS FEB RAS 7787
SC			partly broken, top separated		apical whorls compressed	juvenile	broken after SEM study
ALC	plicate	plicate	non-plicate, thin	non-plicate, thin	thin lip	undeveloped lip	non-plicate, thin
SH	1.72	1.75	1.75	1.63	1.75	1.43	1.69
HLWH	1.13	0.6	0.57	0.58	0.55	0.45	0.69
PH	0.78	0.9	0.9	0.85	0.95	0.68	0.79
SW	1.19	1.30	1.35	1.15	1.25	1.05	1.14
PD	0.69	0.63	0.73	0.65	0.8	0.65	0.69
W	5.65	5.75	5.75	5.7	5.5	5.1	5.7
SA	68.6	68.0	72.0	68.0	76.0	73.5	66.5

##### Etymology.

The new species is named after Nodong cave, the type locality.

##### Type locality.

South Korea, North Chungcheong Province, Danyang County, Nodong cave, 36°57.186'N, 128°22.938'E, alt. ca. 270 m, moist muddy walls in cave.

##### Distribution.

Only known from the type locality.

##### Ecology.

Suggested mix of volcanic elements in cave mud of Nodong cave.

##### Conservation status.

A cursory search through the Internet indicates that the region harboring caves encompassed within the administrative boundaries of Danyang County is greatly threatened. Due to the abundance of limestone in the area, cement factories are big industries there. Of more immediate threat, however, is the frequent human traffic that the caves of Nodong, Gosu and Cheondong receive in light of their popularity as tourist attractions. To exacerbate concerns, a newly built stairway into the deepest, darkest sections of the cave has made Nodong more accessible (Lee 2012). Since *Koreozospeum nodongense* sp. n. is known to live in only one locality and the area is potentially declining due to human encroachment, this species is Critically Endangered (CR) under IUCN criteria (IUCN 2014).

##### Remarks.

*Koreozospeum nodongense* sp. n. appears to be polymorphic in regards to the configuration of a plicate versus non-plicate apertural lip (side view). This elaboration of the lip was apparent in two shells (NMBE 534197/1; ZIN RAS 1) of the five examined shells (1 juvenile with undeveloped lip). We have little doubt that the plicate and non-plicate specimens co-occurring at Nondong cave are conspecific. Prozorova et al. (2011) initially examined the paratype specimen (IBSS FEB RAS 7787) using SEM (Fig. 4). This work revealed microstructural pitting on the protoconch in sync with the concentric pitting pattern reported by Jochum (2011) as a consistent character for the worldwide members of the extant Carychiidae. Protoconch pitting is also known in Eastern European carychiid fossils examined via SEM (Strauch 1977, Stworzewicz 1999, Harzhauser et al. 2014a, 2014b, Jochum et al. 2015). In congruence with the findings of Prozorova et al. (2011), the fragments of *Koreozospeum nodongense* sp. n. here show tightly spaced irregular spiral striae of densely interconnected pits with some occasional, non-pitted patchy zones over the entire teleoconch (Fig. 9). This dense pattern of total teleoconch pitting is also found in *Zospeum isselianum* Pollonera, 1887 and *Zospeum bellesi* Gittenberger, 1973 (Jochum, unpublished data).

The SEM-EDX analysis (Fig. 10A–B) of the surface structure located in the central zone of the fragment edge and the internal surface of the shell shows varying concentrations of the same elements, including calcium (Ca), aluminum (Al), silicon (Si), oxygen (O) and carbon (C) for these two separate regions of the shell. A band (Fig. 11A) of likely volcanic origin of the mud (i.e. lava and alkali basalt) is indicated on the surface of one of the shell fragments. This band contains fractions of the elements calcium (Ca), aluminum (Al), silicon (Si), oxygen (O), carbon (C), iron (Fe), zinc (Zn), chromium (Cr), chlorine (Cl), magnesium (Mg) and potassium (K). The non-banded region of shell (Fig. 11B) shows varying concentrations of calcium (Ca), aluminum (Al), silicon (Si), oxygen (O), carbon (C) and iron (Fe).

**Figure 8.**
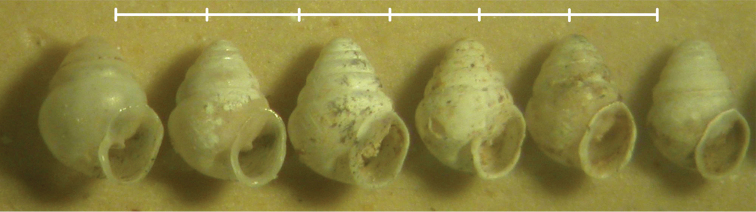
*Koreozospeum nodongense* sp. n. paratype shells (ZIN RAS 1). Scale bar increments 1 mm.

**Figure 9.**
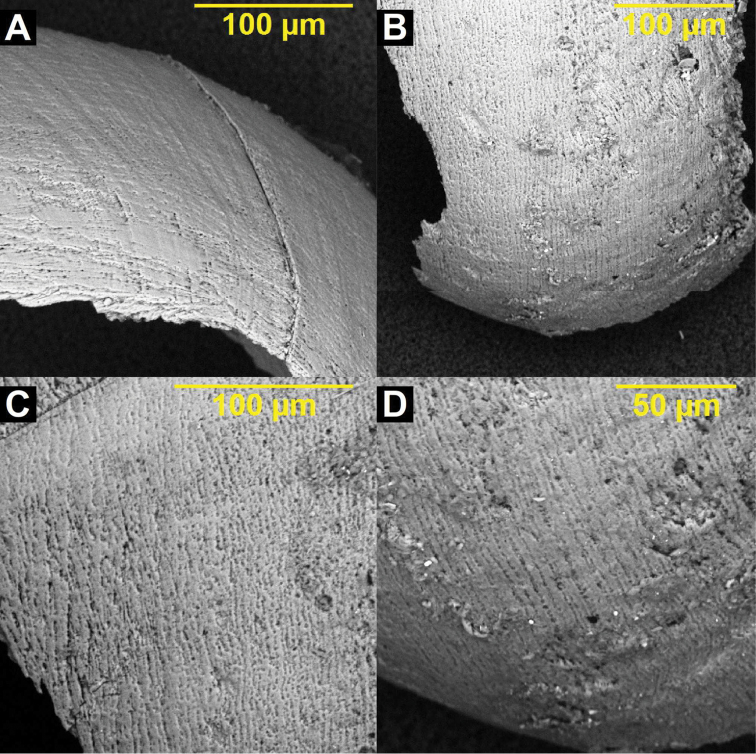
SEM of microstructure pattern on surface of teleoconch fragments of *Koreozospeum nodongense* sp. n. paratype (NMBE 534361/2) showing tightly spaced irregular spiral striae of densely interconnected pits.

**Figure 10.**
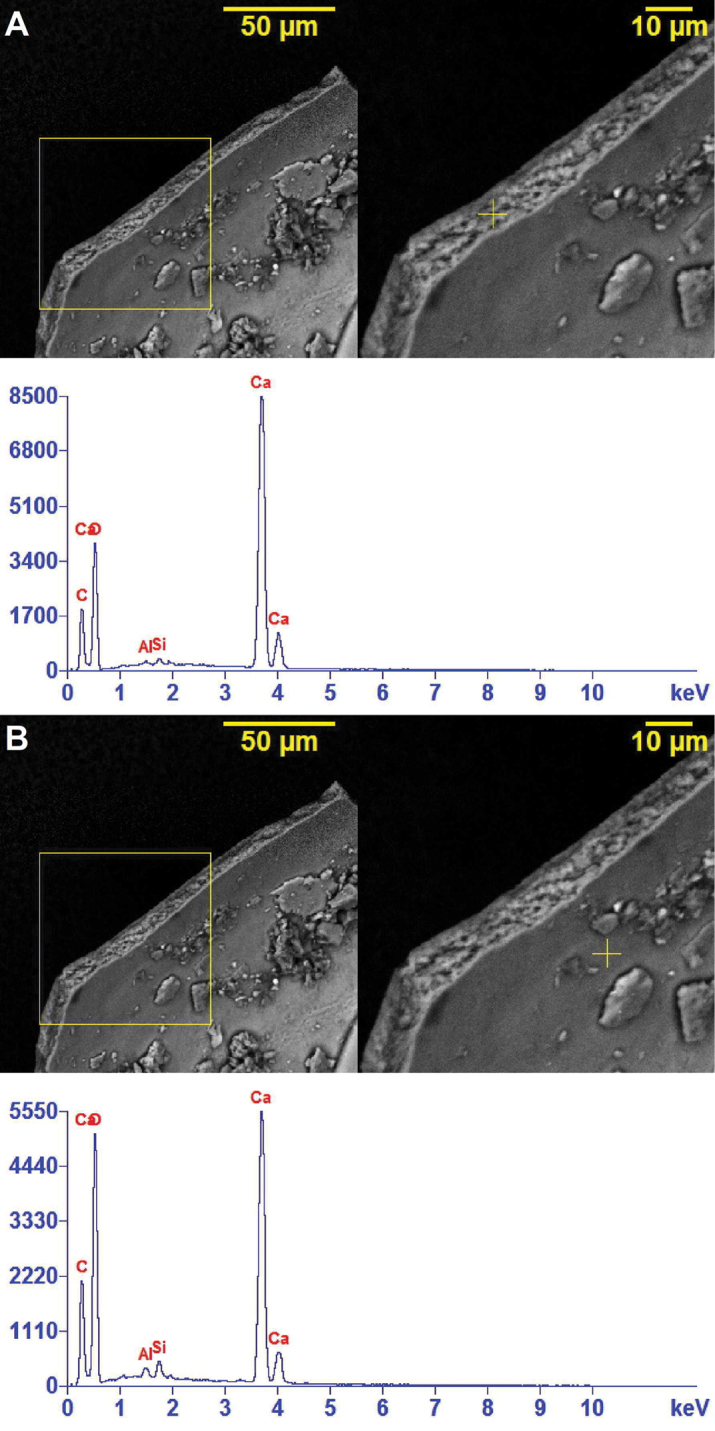
SEM-EDX spectroscopic images showing the spectrum of the elemental content in two regions of the inner shell of *Koreozospeum nodongense* sp. n. paratype (NMBE 534361/2). **A** surface of shell edge **B** the inner layer of the shell. Both regions (yellow +) show the presence of calcium (Ca), aluminum (Al), silicon (Si) and carbon (C).

**Figure 11.**
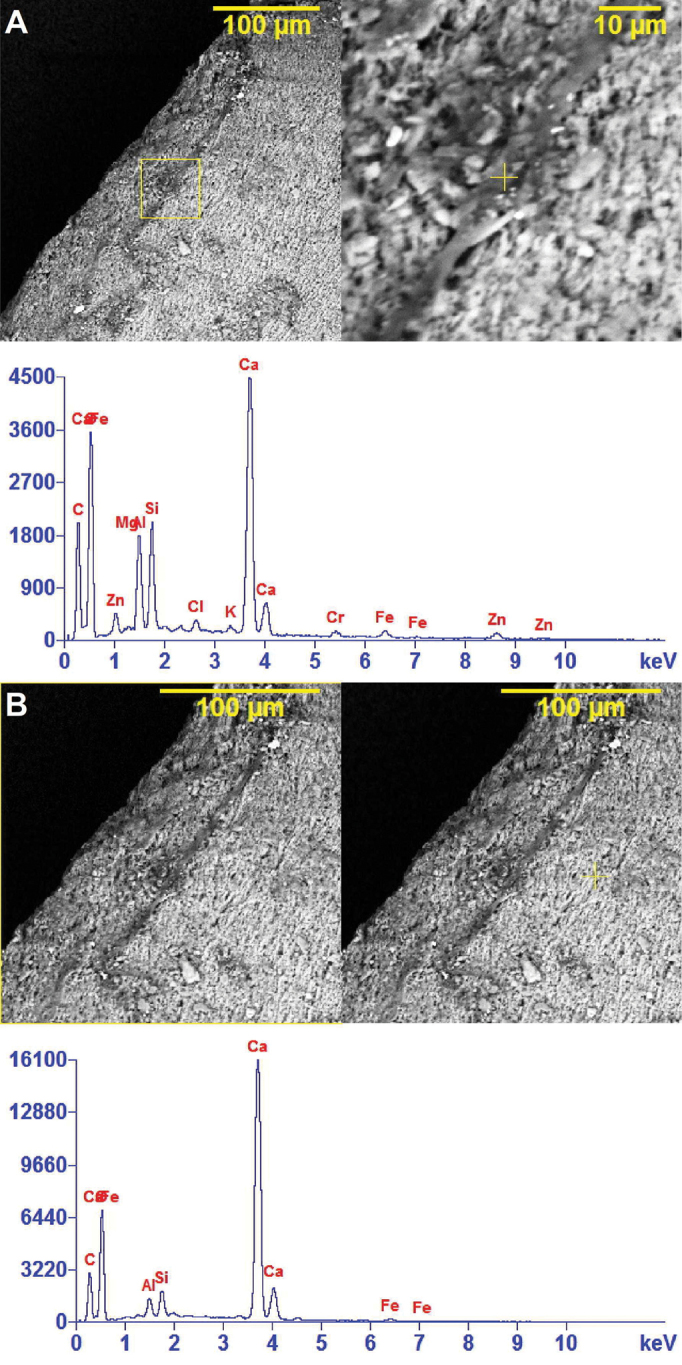
SEM-EDX spectroscopic images showing the elemental spectrum of two regions (yellow +) on the outside surface of the shell of *Koreozospeum nodongense* sp. n. paratype (NMBE 534361/2. **A** band of compacted residue (sediment?) on the surface of the shell containing calcium (Ca), aluminum (Al), silicon (Si), oxygen (O), carbon (C), Iron (Fe), zinc (Zn), chromium (Cr) and potassium (K) **B** region to the left of **A** showing presence of calcium (Ca), aluminum (Al), silicon (Si), oxygen (O), carbon (C) and iron (Fe).

Interestingly for *Koreozospeum nodongense* sp. n. is that the trace elements, aluminum (Al) and silicon (Si), might potentially be involved in the biomineralization process of the shell matrix. It is not clearly discernable whether or not they are intrinsic to the shell or represent contaminants from the substrate. Further study, independent of this work, involving major- and trace element analysis coupled with isotope geochemical analysis might suggest the relatively large variability of elements found in our SEM-EDX analyses to be due to the heterogeneous nature of different magmas mixing at different stages of their evolution in historic volcanic eruptions in South Korea (Brenna et al. 2012). Eroded lava particulates and ash may well constitute the sediment overlying the Ordovician limestone of Danyang County.

## Supplementary Material

XML Treatment for Koreozospeum

XML Treatment for Koreozospeum nodongense
